# Comparing the Antimicrobial In Vitro Efficacy of Amoxicillin/Metronidazole against Azithromycin—A Systematic Review

**DOI:** 10.3390/dj6040059

**Published:** 2018-10-20

**Authors:** Manuela Kaufmann, Patrik Lenherr, Clemens Walter, Thomas Thurnheer, Thomas Attin, Daniel B. Wiedemeier, Patrick R. Schmidlin

**Affiliations:** 1 Clinic of Preventive Dentistry, Periodontology and Cariology, Center of Dental Medicine, University of Zurich, CH-8032 Zurich, Switzerland; thomas.thurnheer@zzm.uzh.ch (T.T.); thomas.attin@zzm.uzh.ch (T.A.); patrick.schmidlin@zzm.uzh.ch (P.R.S.); 2 Private Practice, Zahnmedizin Wiesental, CH-9100 Herisau, Switzerland; patrik.lenherr@zahnmedizin-wiesental.ch; 3 Department of Periodontology, Endodontology and Cariology, University Centre for Dental Medicine, University of Basel, CH-4056 Basel, Switzerland; clemens.walter@unibas.ch; 4 Statistical Services, Center of Dental Medicine, University of Zurich, CH-8032 Zurich, Switzerland; daniel.wiedemeier@zzm.uzh.ch

**Keywords:** systematic review, azithromycin, macrolide, amoxicillin, metronidazole, antimicrobial, in vitro

## Abstract

On account of its proven clinical efficacy, the combination of systemically administered amoxicillin and metronidazole is frequently adjuncted to non-operative periodontal therapy and well documented. Potential drawbacks of this regimen, e.g., side effects and problems with the compliance, led to an ongoing search for alternatives. Azithromycin, an antibiotic extensively used in general medicine, has recently found its niche in periodontal therapy as well. This systematic review aimed to analyze the in vitro antimicrobial efficacy of amoxicillin plus metronidazole versus azithromycin. For this purpose, a systematic literature search was performed, and studies published up to 29 March 2018 referenced in Medline, Embase, Cochrane, and Biosis were independently screened by two authors. An additional hand search was performed and studies focusing on the evaluation of in vitro antimicrobial efficacy of amoxicillin + metronidazole or azithromycin on bacteria from the subgingival biofilm were included. English and German language research reports were considered. From 71 identified articles, only three articles were eligible for inclusion. These studies showed heterogeneity in terms of analytical methods and strains explored. However, all studies used multispecies biofilm models for analysis of the antimicrobial activity. Unanimously, studies reported on more pronounced antimicrobial effects when applying the combination of amoxicillin + metronidazole, compared to azithromycin. Based on the few studies available, the combination of amoxicillin + metronidazole seemed to display higher antimicrobial efficacy in vitro than azithromycin.

## 1. Introduction

Periodontitis is a highly prevalent infectious disease leading to an inflammatory host response, destruction of tooth supporting tissues, and finally tooth loss if left untreated [1]. In this context, oral bacteria colonize the tooth surfaces in the form of complex biofilms. Failure to control or eliminate pathogenic biofilms by means of adequate individual and/or professional oral hygiene measures, leads to bacterial shifts and dysbiosis. Clinically, the transition from gingivitis to periodontitis may occur [2]. Given this microbial etiology, the application of systemic antibiotics as an adjunctive therapy to mechanical biofilm management has been explored for various disease entities [3].

The clinical efficacy of the combination of amoxicillin (AMX) and metronidazole (MTZ), adjuncted to mechanical treatment, has been extensively studied and is well documented [4]. So far, distinct antibiotic medication superiority over AMX + MTZ in combination has not been shown, neither in vivo nor in vitro studies [5]. However, some clinical drawbacks, such as adverse side-effects, difficulties in maintaining patient compliance, the high dose prescribed, and duration of therapy/intake of drugs or alcohol incompatibility have shown to complicate or limit the outcome with these drugs [3].

Lately, the macrolide antibiotic azithromycin (AZM) has been proposed as a possible alternative [6]. This antibiotic is interesting for periodontal therapy due to several potential benefits, including a broad antimicrobial spectrum, anti-inflammatory activity, a lower intake dosage and duration/frequency of intake [2]. Unfortunately, comparative data on a direct clinical comparison between AZM and the combination of AMX + MTZ are still scarce and ongoing. However, a recent systematic review found clear evidence for AZM as a second choice alternative to the combination of AMX + MTZ in chronic periodontitis patients [6].

The clinical application of drugs needs to be supported by the evidence from in vitro analyses, which corroborate the adequate antimicrobial spectrum. However, comparative laboratory data from in vitro antimicrobial testing of AZM and AMX + MTZ have not been systematically assessed yet. Therefore, this systematic review aimed to evaluate the in vitro antimicrobial efficacy of AMX + MTZ versus AZM. We hypothesized that both antibiotic therapies were equally effective in eliminating in vitro, the growth of bacteria associated with periodontal disease.

## 2. Materials and Methods

This study matched the PRISMA (Preferred Reporting Items for Systematic Reviews and Meta-Analyses) guidelines for systematic reviews [7]. The focused question was adjusted according to the PICO (Population, Intervention, Comparator, Outcome) criteria for comparing laboratory studies as follows [8]:

“What is the in vitro antimicrobial efficacy of amoxicillin + metronidazole compared to azithromycin when targeting periopathogenic bacteria?”

### 2.1. Search Strategy

A literature search was performed using the U.S. National Library of Medicine (Medline), Excerpta Medical Database (Embase), Biosis Previews Database, and the Cochrane Central Library. Articles were included up to and including 29 March 2018.

The following terms were explored: (azithromycin) OR (zithromax) AND (metronidazole AND amoxicillin) OR (“van winkelhoff”) AND (periodontitis) OR (periodontal) including the according MeSH terms respectively.

### 2.2. Study Selection

Two reviewers (MK, PL) independently screened titles and abstracts found in the electronic search and assessed them in a first step to possibly include them into the review. All potentially eligible studies were ordered and their full texts were assessed. Studies were included if published in English or in German. Disagreement between the reviewers was resolved by discussion.

### 2.3. Eligibility Criteria for Studies

Only in vitro studies were considered.

**Figure 1:**
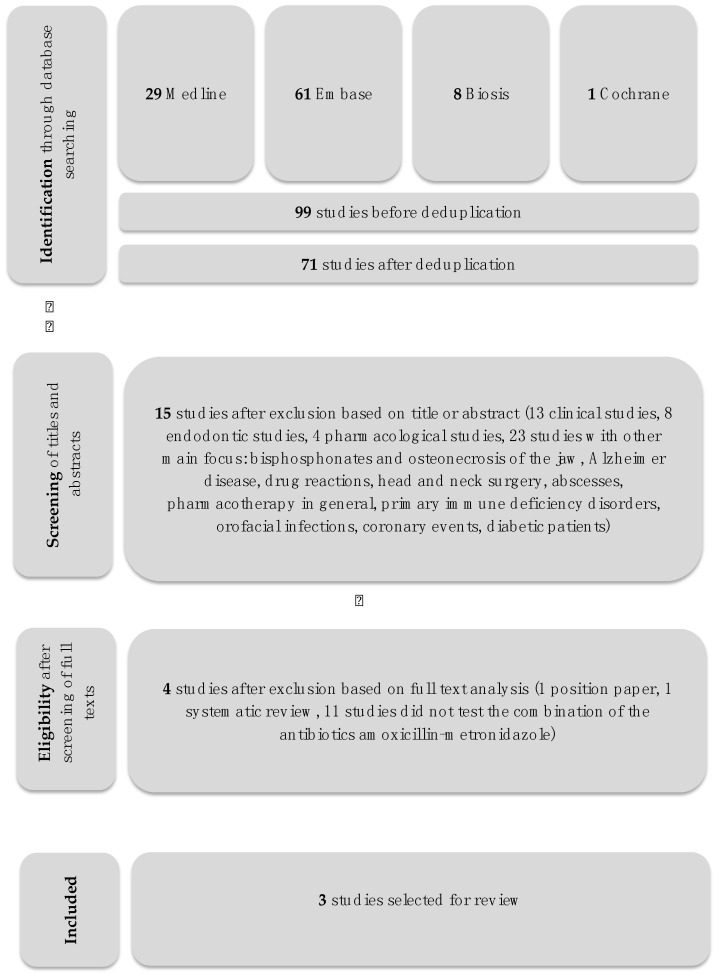
Study selection flowchart.

Figure 1 represents a PRISMA flowchart of the selection process of the included studies.

### 2.4. Data Extraction

Excluded articles were classified hierarchically and explanations for exclusion were provided individually.

## 3. Results

### 3.1. Search and Screening

From 71 titles identified through the electronic search, sixty-seven articles were excluded (Figure 1, Table 1). The main reasons for exclusions were: In-vitro studies not testing the desired antibiotic regimen (12 studies), other study designs (total 23 studies: 2× surveys, 1× narrative review, 2× focus on head and neck infections, 3× testing of only one specific antibiotic, 1× evaluation of an odontogenic infection, 1× focus on HSV-1 and Alzheimer disease, 1× abscesses, 1× HIV, 1× vaccines and PDT, 1× critical review on antimicrobial treatments in general, 1× coronary events, 1× orofacial infections, 2× osteonecrosis of the jaw and bisphosphonates, 2× antibiotic resistance in general, 1× diabetic patients, 1× review on antibiotic prophylaxis, 1× immunology in general) or other topics within the dental field (20 clinical, 8 endodontic and 5 pharmacological, respectively). The inter-rater agreement was 100%. 

Although one of the screened study met all inclusion criteria [9], the full text was not available despite contacting the respective authors and libraries. Therefore, the study had to be excluded. The remaining three full articles were again separately and independently assessed by both reviewers. Finally, three publications were included in the systematic analysis.

### 3.2. Experimental Methods in the Evaluated Studies

The in vitro experiments in this review were assessed for methodological heterogeneity. The experiments are briefly described below.

Belibasakis & Thurnheer [14] employed an in vitro subgingival biofilm model with 10 species [81]. After culturing the bacteria for 40.5 h, the biofilms were exposed to the following antibiotics for another 24 h at concentrations detected in the pocket environment following systematic administration (15 mg/L MTZ, 15 mg/L AMX, a combination of 15 mg/L MTZ + 15 mg/L AMX, 2 mg/L doxycycline and 10 mg/L AZM). Bacterial counts and final concentrations of the antibiotics in the culture media were then measured. Three independent experiments were performed for control as well.

Ong and co-workers [46] evaluated the efficacy of AZM on mono- and polymicrobial biofilm formations consisting of *P. gingivalis*, *T. denticola,* and *T. forsythia* in comparison to AMX + MTZ in combination in vitro. The antibiotics were dispersed using deionized water and final antibiotic concentrations in the range between 0.01–100 mg/L were tested in the respective supernatants of the microbial cultures. Monitoring the growth for 48 h, they measured absorbance at a wavelength of 620 nm (AU_620_) using a microplate reader. The minimum inhibitory concentration (MIC) and the minimal biofilm inhibitory concentration (MBIC) for the antibiotics were calculated. 

Soares et al. [65] tested the antimicrobial effects of AZM and the combination of AMX + MTZ on a polymicrobial biofilm model with 35 subgingival bacterial species, including *S. oralis*, *F. nucleatum*, *P. gingivalis*, *P. intermedia*, *A. actinomycetemcomitans,* and *T. forsythia.* A 2,3,5-triphenyltetrazolium chloride (TTC) was used in this study to discern metabolically active and inactive cells. The white substrate was enzymatically reduced to red 1,3,4-triphenylformazan (TFP) by living bacterial cells, as a result of dehydrogenase activity. By reading changing substrate color through fluorescence spectrophotometry, the reduction rate was registered. To measure the undergoing metamorphosis of the biofilms, the remaining pegs were rinsed in solution twice and moved onto 9 plates where the TTC conversion was taken at 485 nm. After the TTC assay, the pegs were washed, extracted from the cover and transferred to Eppendorf tubes (final solution). Using checkerboard DNA–DNA hybridization technique [82], the samples then were individually analyzed. The database from a previous study by the same group was examined to obtain reference values for the composition of in vivo biofilms. The consistency of the microbial profiles was tested across and between the nine in vitro biofilm samples, and the in vivo reference values by using a so-called minimum similarity coefficient. The total DNA probe count was calculated for each species in each in vitro biofilm sample, and for the mean reference values for in vivo biofilms. Once the minimum similarity value for each species in a pair of samples was calculated, the values were summed to give a summary measurement for the entire microbial community.

For each time point, the three negative-control optical density (OD) values were averaged and each assay result was divided by this number, which yielded the proportion of activity remaining in the presence of the antibiotic. This proportion was then subtracted from 1 and multiplied by 100 to derive the percent inhibition.

At chosen time points (12, 24, 36 h), four different concentrations of the antibiotics (1:1, 1:3, 1:9, 1:27) were tested. The 1:27 dilutions at the 36 h time point were considered to be the primary analysis, as this concentration was likely to be closest to that achieved in periodontal pockets when antibiotics are administered systemically. The analyses for the remaining dilutions and time points were considered exploratory.

### 3.3. Antimicrobial Results

AMX + MTZ reduced the biofilm 27% more than AZM [14]. The metabolic activity was reduced 84% through AMX + MTZ whereas only by 17% through AZM [65]. The MIC and MBIC of AMX + MTZ in combination was found to be almost 10-fold lower than the one of AZM [46].

Taken together, all of the studies reported on more pronounced antimicrobial effects (biofilm reduction, growth inhibition or reduction of metabolic activity) of AMX + MTZ compared to AZM in in vitro biofilm models.

Table 2 Comparison of the three studies selected in the review.

## 4. Discussion

The purpose of the present review was to evaluate the available literature for the in vitro antimicrobial efficacy of AZM, compared to the combination of AMX + MTZ. Based on the few studies available, the combination of AMX + MTZ was unequivocally shown to have a higher antimicrobial efficacy in vitro, compared to AZM. 

Despite these results and within the main limitation of this review, namely a low number of underlying studies supporting this finding, antibiotics have a specific antimicrobial range of efficacy which has to be taken into account. The range of indications and the use of these antibiotics need to be matched with the suspected microbes, but should also be as broad as possible. 

In vitro biofilm models have been proposed as a means to examine the higher tolerance to antimicrobials that this mode of growth confers to bacteria [83]. It has been argued that, due to greater tolerance to antimicrobials, that MICs calculated using bacterial cells grown planktonically would bear little relevance to in vivo situations [2]. The higher tolerance of biofilms to antimicrobials has also led periodontists to recommend that the use of these agents be accompanied or preceded by mechanical disruption of the subgingival biomass.

In medicine AZM is extensively used as medication for a widespread spectrum of infections [2]. It is an industrially processed analogue of erythromycin with a supplementary nitrogen atom in the macrocyclic lactone ring [84]. In AZM, structural stability is provided through the extra nitrogen atom. Compared with erythromycin, tissue penetration is optimized, toxicity is low, and half-life is almost three days [85]. As the course of administration is short along with the list of side effects as well, patient compliance is excellent [86]. In susceptible organisms, AZM reversibly hinders bacterial protein synthesis by addressing the 23S ribosomal RNA of the 50S ribosomal subunit [87]. Macrolides decrease bacterial adhesion, resulting in minified biofilm formation. This process is dose-dependent and occurs even at very low macrolide concentrations [88]. AZM has bacteriostatic effects against a wide spectrum of bacteria in vitro and is particularly effective against gram-negative anaerobic bacteria [89]. Gingival crevicular fluid (GCF) concentrations of AZM, following a 500 mg oral dose, have been shown to reach up to 7–8 mg/L. Levels of AZM in serum are 40-fold lower [24]. The authors attributed this to cells of peripheral tissues which accumulate AZM actively. Significant immunomodulatory effects of AZM have been observed. In vitro concentrations of AZM lower than MIC could significantly inhibit quorum-sensing signals and biofilm formation of *P. aeruginosa* [25]. Beneficially, AZM may possibly decrease proinflammatory cytokine production [90]. It must be noted that such mechanisms were not investigated in the included articles, which focused on antimicrobial actions only. Therefore, additional working actions may increase the efficacy of AZM, which has to be compared in a respective systematic evaluation of clinical studies. This is another shortcoming of this study—if interpreted as a singular antimicrobial evaluation, as it ignores additional modes of action as mentioned above.

AMX is a bacteriolytical lactame-antibiotic with a wide spectrum and a half-life of 1 to 1.5 h. MTZ is, as a nitroimidazole, especially effective against anaerobes and protozoa with a half-life of 6 to 7 h. The simultaneous administration of these two medications has gained increased significance over the last two decades. Both active substances in combination cover most of the aerobic and anaerobic bacteria. Furthermore, they overlap in their effect on facultative bacteria. The combination of MTZ + AMX has gained recognition mostly due to its efficacy against *A. actinomycetemcomitans*, a periodontal pathogen closely associated with the etiology of rapid periodontitis progression [91]. A possible explanation for the synergistic effects of MTZ + AMX may be increased uptake of MTZ in the presence of AMX, as has been described for *A. actinomycetemcomitans* in Reference [28]. AMX + MTZ have been prescribed for more than three decades; their effects and side-effects are well documented. The most common adverse reactions to AMX are allergic, often mild forms, limited exanthema on the head and neck. Heavy reactions may cause swelling of joints. Anaphylactic reactions are possible with highly sensitive patients. Possible side-effects of MTZ are nausea, headache, lack of appetite, diarrhea or metallic taste, seldom rashes [4]. Additionally, this combi-cocktail requires increased patient-compliance with the intake of two tablets, three times daily, over a period of seven days [6]. In case of penicillin allergy, the combination of MTZ (500 mg) and Ciprofloxacin (250 mg) twice daily is recommended [92].

For a number of putative periodontal pathogens, the MIC is shown to be below the antibiotic concentrations achievable in GCF [93]. The concentration in the GCF rises three days after administration of 500 mg AMX at 14 g/mL [94], and two days after administration of 500 mg MTZ at 13 g/mL [95]. Administration of 500 mg AZM, followed by 250 mg, results in 7.5 g/mL after two days and 2.5 g/mL after fifteen days [96]. “The antibiotic concentration in GCF should be considerably higher than the MICs indicated by their in vitro efficacy to be effective within the environment” [34]. 

At a concentration of 15 mg/L, Reference [14] reported that total cell numbers were reduced through AMX + MTZ in combination, and significantly reduced *P. gingivalis* numbers, whereas AZM, at a concentration of 10 mg/L, reduced total cell numbers at less than 0.5 log. However, the clinical efficacy might be underestimated as in vivo the microbial load of *P. gingivalis* might be markedly lower compared to those reported in the three studies.

Soares et al. [65] stated that they were able to consistently recover 35 of the initial 40 bacteria used in their biofilm model. The missing bacteria, especially Gram-negative *Prevotella* species, might explain the increased tolerance of the in vitro biofilm to MTZ, which specifically targets strict anaerobic Gram-negative bacteria. Furthermore, *Treponema* species were also excluded from the in vitro model, due to difficulties in growing these strict anaerobes. Despite this, biofilm models provide a more relevant test scenario for testing the efficacy of antibiotics as opposed to bacterial cultures in a planktonic state, for which the MICs are purportedly lower.

By using only three bacterial species as in the study by Ong [46], the complexity of the polymicrobial biofilms accomplished with subgingival plaque might not have been completely represented. Still, these three species close to the gingival epithelium, forming a bilateral bacterial neighborhood, are affiliated with disease severity and progression [88]. However, the Zurich biofilm model does not include host immune cells. These cells in the periodontal pocket environment could support the effect of antibiotics. Two of the studies used bacterial strains taken from the American Typing Collection (ATCC). The Zurich group [14] took bacterial strains from their own cultivation (OMZ: Institute for Oral Biology, Section for Oral Microbiology and General Immunology, University of Zurich, Zurich, Switzerland).

All authors of the three studies agreed that it was difficult to reconcile longitudinal studies with in vitro antimicrobial testing. The subgingival biofilm model used in the Soares study [65] might simulate the most realistic clinical situation yet. However, in general, in vitro oral multispecies biofilm models all suffer from one or more limitations and are comprised of only up to 5 or 10 species [89].

## 5. Conclusions

This review of the literature does not allow drawing definitive conclusions regarding the clinical efficacy of AMX + MTZ in combination versus AZM, due to the small number of studies available for inclusion and the very different test protocols. In addition, only antimicrobial assessment was performed, neglecting other potential action models. Whilst the combination of AMX + MTZ performed in 3 out of 3 studies better and seemed to have a higher antimicrobial efficacy in vitro as compared to AZM, further studies are required to evaluate the comparative laboratory susceptibility and the clinical relevance of AZM in particular.

## Figures and Tables

**Table 1:** Excluded studies. The reason for exclusion was arranged in categories pharma (for pharmacological studies), endo (for endodontic studies), other (for studies not addressing the research question), in-vitro (for in-vitro studies) or clin (for clinical studies).

	Reference	Category	Exclusion Criteria
**1)**	*2004 Position paper* [10]	pharma	description of systemic antibiotics in periodontics in general, not specifically in vitro
**2)**	*2017 Alattas et al.* [11]	endo	reporting about prescription of antibiotics usus in southern Saudi Arabia focusing endodontic pathology
**3)**	*2016 Barbosa-Ribeiro et al.* [12]	endo	focusing antimicrobial susceptibility after failure of endodontic treatment
**4)**	*2013 Bartold et al.* [13]	other	focusing only AZM
**5)**	*2014 Belibasakis & Thurnheer* [14]	in-vitro	
**6)**	*2013 Brook* [15]	other	focus on head and neck infections in general rather than periodontal aspects
**7)**	*2015 Brook* [16]	other	focus on head and neck infections in general rather than periodontal aspects
**8)**	*2000 Carrasco et al.* [17]	in-vitro	the combination of the antibiotics AMX-MTZ wasn’t tested
**9)**	*2015 Chopra et al.* [18]	other	focus on cutaneous adverse drug reactions
**10)**	*1995 Coulaud* [19]	other	focusing only AZM
**11)**	*2016 Dakic et al.* [20]	clin	clinical study, AZM wasn’t tested, systematic review and meta-analysis
**12)**	*2015 Ercan et al.* [21]	clin	clinical study, patients with chronic periodontitis
**13)**	*2001 Feik et al.* [22]	in-vitro	the combination of the antibiotics AMX-MTZ wasn’t tested
**14)**	*1997 Fresnadillo et al.* [23]	clin	clinical study
**15)**	*2014 Garg et al.* [24]	other	survey
**16)**	*2004 Greenstein et al.* [25]	clin	clinical study
**17)**	*2015 Harris et al.* [26]	other	focus on HSV-1 and Alzheimer disease
**18)**	*2003 Hernandez-rizzo* [9]	in-vitro	full text wasn’t available until data
**19)**	*2012 Herrera et al.* [27]	clin	clinical study
**20)**	*2008 Isla et al.* [28]	other	focus on odontogenic infections in general rather than periodontitis
**21)**	*2003 Jacinto et al.* [29]	endo	focus on analysis of infected root canals
**22)**	*2011 Japoni et al.* [30]	in-vitro	the combination of the antibiotics AMX-MTZ wasn’t tested
**23)**	*2005 Jaramillo et al.* [31]	other	clin and in vitro, but focus on abscesses
**24)**	*2016 Jentsch et al.* [32]	clin	clinical study
**25)**	*2015 Keestra et al.* [33]	clin	clinical study, systematic review and meta-analysis
**26)**	*2007 Kuriyama et al.* [34]	in-vitro	the combination of the antibiotics AMX-MTZ wasn’t tested
**27)**	*2013 Kuruvilla et al.* [35]	other	focus on primary immune deficiency disorders
**28)**	*2011 Leszczyńska et al.* [36]	pharma	updated review, focus on periodontal pharmacotherapy in general
**29)**	*2009 Liu et al.* [37]	other	focus on vaccines and PDT
**30)**	*1999 Loesche* [38]	other	critical review, focus on antimicrobial treatment of periodontal disease
**31)**	*2007 Maestre et al.* [39]	in-vitro	the combination of the antibiotics AMX-MTZ wasn’t tested
**32)**	*2012 Mahajan et al.* [40]	endo	focus on management of endodontic infections
**33)**	*2007 Mattina* [41]	other	focus on Clarithromycin
**34)**	*2018 McGowan et al.* [42]	clin	clinical study, AZM wasn’t tested, systematic review and meta-analysis of RCTs
**35)**	*2011 Mouratidou et al.* [43]	in-vitro	the combination of the antibiotics AMX-MTZ wasn’t tested
**36)**	*2013 Muniz et al.* [44]	other	focusing only AZM
**37)**	*2003 Murillo* [45]	clin	focus on orofacial infections
**38)**	*2017 Ong et al.* [46]	in-vitro	
**39)**	*2007 Paju et al.* [47]	other	focus on coronary events
**40)**	*2017 Palappallil et al.* [48]	pharma	focus on adverse drug reactions
**41)**	*2016 Papathanasiou et al.* [49]	other	a survey of periodontists in the US
**42)**	*2015 Parenti et al.* [50]	other	narrative review with focus on endothelial dysfunction
**43)**	*2011 Parnham* [51]	other	focus on immunology in general
**44)**	*2014 Rams et al.* [52]	in-vitro	the combination of the antibiotics AMX-MTZ wasn’t tested
**45)**	*2012 Ramu et al.* [53]	other	practice review on antibiotic prophylaxis
**46)**	*2015 Ranganathan et al.* [54]	clin	clinical study
**47)**	*2003 Rolim De Sousa et al.* [55]	endo	bacteriological study of root canals associated with periapical abscesses
**48)**	*2005 Ryan* [56]	clin	clinical study
**49)**	*2016 Saleh et al.* [57]	clin	clinical study, patients with chronic periodontitis
**50)**	*2015 Santos et al.* [58]	other	focus on diabetic patients, systematic review
**51)**	*2010 Segura-Egea et al.* [59]	endo	focus on management of endodontic infections amongst Spanish oral surgeons
**51)**	*2009 Serrano et al.* [60]	other	focus on antibiotic resistance of periodontal pathogens
**52)**	*2012 Sgolastra et al.* [61]	clin	clinical study, AZM wasn’t tested, systematic review and meta-analysis
**53)**	*2011 Shannon et al.* [62]	other	focus on Bisphosphonates and osteonecrosis of the jaw
**54)**	*2017 Shivi et al.* [63]	clin	clinical study
**55)**	*2013 Siqueira et al.* [64]	other	focus on abscesses
**56)**	*2015 Soares et al.* [65]	in-vitro	
**57)**	*2011 Somma et al.* [66]	endo	focus on endo and general health
**58)**	*2013 Sousa et al.* [67]	endo	focus on antimicrobial susceptibility pattern of infected root canals
**59)**	*2018 Souto et al.* [68]	clin	clinical study, focus on diabetic subjects, systematic review and meta-analysis
**60)**	*2004 Sweeny et al.* [69]	other	focus on antibiotic resistance in the dental practice
**61)**	*2001 Tarullo et al.* [70]	clin	focus on Helicobacter pylori
**62)**	*2014 Teughels et al.* [71]	clin	clinical study, patients with aggressive periodontitis
**63)**	*2007 Tomas et al.* [72]	in-vitro	the combination of the antibiotics AMX-MTZ wasn’t tested
**64)**	*2007 Van Den Wyngaert et al.* [73]	other	focus on osteonecrosis of the jaw and bisphosphonates
**65a),65b)**	*1999, 2000 van Winkelhoff et al.* [74,75]	in-vitro	the combination of the antibiotics AMX-MTZ wasn’t tested
**66)**	*2005 van Winkelhoff et al.* [76]	in-vitro	the combination of the antibiotics AMX-MTZ wasn’t tested
**67)**	*2012 Veloo et al.* [77]	in-vitro	the combination of the antibiotics AMX-MTZ wasn’t tested
**68)**	*2005 Voils et al.* [78]	clin	clinical study
**69)**	*2013 Zandbergen et al.* [79]	clin	clinical study, AZM wasn’t tested, systematic review
**70)**	*2016 Zhang et al.* [80]	clin	clinical study, the combination of the antibiotics AMX-MTZ wasn’t tested, meta-analysis of RCTs

**Table 2:** Comparison of the three studies.

Reference	Tested Outcome Parameters	Antibiotic	Results	Difference (Δ parameter:AMX/MTZ - AZM)
*2014 Belibasakis and Thurnheer* *Validation of antibiotic efficacy on in vitro subgingival biofilms.* [14]	Log_10_ Reduction (TBC) Reduction of biofilm in %	Azithromycin	0.4 log 60%	Δ 0.5 log Δ 27%
		Amoxicillin/ Metronidazole	0.9 log 87%	
*2017 Ong et al.* *Effect of azithromycin on a red complex polymicrobial biofilm.* [46]	MIC MBIC	Azithromycin	MIC 1.52 mg/L MBIC 10.6 mg/L	Δ MIC 1.35 mg/L Δ MBIC 9.3 mg/L
		Amoxicillin/ Metronidazole	MIC 0.17 mg/L MBIC 1.3 mg/L	
*2015 Soares et al.* *Effects of azithromycin, metronidazole, amoxicillin, and metronidazole plus amoxicillin on an in vitro polymicrobial subgingival biofilm model. Antimicrobial agents and chemotherapy.* [65]	Reduction of metabolic activity in %	Azithromycin	17%	Δ 67%
		Amoxicillin/ Metronidazole	84%	
